# Genetic Diversity, Multidrug Resistance, and Virulence of *Citrobacter freundii* From Diarrheal Patients and Healthy Individuals

**DOI:** 10.3389/fcimb.2018.00233

**Published:** 2018-07-10

**Authors:** Liyun Liu, Daoli Chen, Liqin Liu, Ruiting Lan, Shuai Hao, Wenjie Jin, Hui Sun, Yiting Wang, Ying Liang, Jianguo Xu

**Affiliations:** ^1^State Key Laboratory of Infectious Disease Prevention and Control, National Institute for Communicable Disease Control and Prevention, Chinese Center for Disease Control and Prevention, Beijing, China; ^2^Collaborative Innovation Center for Diagnosis and Treatment of Infectious Diseases, Zhejiang, China; ^3^Maanshan Center for Disease Control and Prevention, Ma'anshan, China; ^4^School of Chemistry and Biological Engineering, University of Science and Technology Beijing, Beijing, China; ^5^School of Biotechnology and Biomolecular Sciences, University of New South Wales, Sydney, NSW, Australia; ^6^Beijing Advanced Innovation Center for Food Nutrition and Human Health, Beijing Engineering and Technology Research Center of Food Additives, Beijing Technology and Business University, Beijing, China

**Keywords:** *Citrobacter freundii*, multilocus sequence typing, multidrug resistance, adhesion, cytotoxicity

## Abstract

**Objectives:** *Citrobacter freundii* is a frequent cause of nosocomial infections and a known cause of diarrheal infections, and has increasingly become multidrug resistant (MDR). In this study, we aimed to determine the genetic diversity, the antimicrobial resistance profiles and *in vitro* virulence properties of *C. freundii* from diarrheal patients and healthy individuals.

**Methods:** 82 *C. freundii* isolates were obtained from human diarrheal outpatients and healthy individuals. Multilocus Sequence Typing (MLST) of seven housekeeping genes was performed. Antimicrobial susceptibility testing was carried out using the disk diffusion method according to the Clinical and Laboratory Standards Institute (CLSI) recommendations. Adhesion and cytotoxicity to HEp-2 cells were assessed. PCR and sequencing were used to identify *bla*_CTX−M_*, bla*_SHV_, *bla*_TEM_, *qnrA, qnrB, qnrS, qnrC, qnrD, aac(6')-Ib-cr*, and *qepA* genes.

**Results:** The 82 *C. freundii* isolates were divided into 76 sequence types (STs) with 65 STs being novel, displaying high genetic diversity. Phylogenetic analysis divided the 82 isolates into 5 clusters. All 82 isolates were sensitive to imipenem (IPM), but resistant to one or more other 16 antibiotics tested. Twenty-six isolates (31.7%) were multidrug resistant to three or more antibiotic classes out of the 10 distinct antibiotic classes tested. Five MDR isolates, all of which were isolated from 2014, harbored one or more of the resistance genes, *bla*_TEM−1_, *bla*_CTX−M−9_, *aac(6')-Ib-cr, qnrS1, qnrB9*, and *qnrB13*. All 11 *qnrB*-carrying *C. freundii* isolates belonged to cluster 1, and one *C. freundii* isolate carried a new *qnrB* gene (*qnrB92*). Six isolates showed strong cytotoxicity to HEp-2 cells, one of which was multidrug resistant.

**Conclusions:** *C. freundii* isolates from human diarrheal outpatients and healthy individuals were diverse with variation in sequence types, antibiotic resistance profiles and virulence properties.

## Introduction

*Citrobacter freundii*, a member of the genus *Citrobacter* within the family *Enterobacteriaceae*, is considered a commensal resident in the intestinal tracts of both humans and animals (Bai et al., 2012). However, *C. freundii* can also cause diarrhea and other infections in humans (Mohanty et al., 2007; Samonis et al., 2009; Bai et al., 2012; Liu et al., 2017a). Some *C. freundii* isolates have acquired virulence traits and caused food poisoning or diarrhea in humans (Bai et al., 2012; Liu et al., 2017a). The main virulence factors found in diarrhea-associated *C. freundii* are toxins, including Shiga-like toxins, heat stable toxins and a cholera toxin B subunit homolog (Bai et al., 2012). In our previous study, we identified a cytotoxic and aggregative *C. freundii* strain which contained a complete type VI secretion system (T6SS) located on a genomic island (GI); we also found two strongly cytotoxic *C. freundii* isolates, which were multidrug resistant, with resistance to ≥3 different classes antibiotics (Liu et al., 2017a).

Antibiotic resistance of *C. freundii* has increased worldwide, and some strains harbored extended-spectrum β-lactamase (ESBL) (Park et al., 2005; Moland et al., 2006; Choi et al., 2007) and plasmid-mediated quinolone resistance (PMQR) determinants (Shao et al., 2011). The prevalence of ESBLs were 4.9–20.6, 0.2–4.6, and 0.9% of *C. freundii* isolates from Korea, Japan and USA, respectively (Park et al., 2005; Moland et al., 2006; Choi et al., 2007). In our previous study, we identified two *C. freundii* isolates harboring a *bla*_TEM−1_ gene (Liu et al., 2017a). As an important PMQR determinant, *qnr* and *aac(6')-Ib-cr* genes have been reported in *C. freundii* (Shao et al., 2011). In Korea, 38.4% of *C. freundii* isolates were found to harbor *qnr* genes (Park et al., 2007). In China, the *qnr* and *aac(6')-Ib-cr* genes were present in 72.8 and 11.6% of clinical *C. freundii* isolates, respectively (Zhang et al., 2012). In our previous study, we found that the *qnr* (*qnrB63* and *qnrS1*) and *aac(6')-Ib-cr* genes were present in 23.1 and 15.4% of *C. freundii* isolates, respectively (Liu et al., 2017a).

The *qnrB* genes constitute the most prevalent and diverse group within the *qnr* family (Ribeiro et al., 2015). Bae et al. have reported that 63.1% of the *qnr* positive clinical *C. freundii* isolates carried *qnrB* (Bae et al., 2010). Our previous study found that two *C. freundii* isolates carried an variant of the *qnrB77* gene (Liu et al., 2017a).

In this study, we analyzed the genetic diversity and antimicrobial resistance profiles of 82 *C. freundii* isolates from diarrheal outpatients and healthy individuals in Maanshan, Anhui Province, China. We investigated the prevalence of *bla*_CTX−M_*, bla*_SHV_, *bla*_TEM_, *qnrA, qnrB, qnrS, qnrC, qnrD, aac(6')-Ib-cr*, and *qepA* genes and determined the adhesion and cytotoxicity to HEp-2 cells of the isolates.

## Materials and methods

### Ethics statement

This study was reviewed and approved by the ethics committee of National Institute for Communicable Disease Control and Prevention, the Chinese CDC. Human fecal specimens were acquired with the written informed consent of the diarrheal patients with the approval of the ethics committee of National Institute for Communicable Disease Control and Prevention, according to the medical research regulations of Ministry of Health (permit number 2007-17-3).

### Citrobacter isolates

Eighty-two *C. freundii* isolates were obtained from 62 diarrheal outpatients and 20 healthy individuals from 2014 to 2016 in Maanshan Anhui Province, China. Fifteen of the 62 diarrheal patient fecal samples harbored other known enteric bacterial or viral pathogens (Table 1). The identity of each isolate was determined using API 20E test strips (bioMérieux, La Balme les Grottes, France) at the time of isolation, and isolates were stored as glycerol stocks at – 80°C. Bacteria were grown in Luria-Bertani (LB) broth or on LB and Mueller–Hinton agar plates (pH 7.4) at 37°C.

**Table 1:** Adherence, cytotoxicity, multidrug resistant, and Genotypes of 82 *C. freundii* Isolates.

**Clusters**	**Isolates**	**Source**	**Year**	**Adhesion**	**LDH**	**MDR**	**ESBLs**	***qnr***	**Other pathogen**
Cluster 1	AH2016011	D	2016	^*^	9.6 ± 2.7	2			
	AH2014010	D	2014	^*^	12.0 ± 1.8	2		*qnrB13*	
	AH2015003	D	2015	–	13.6 ± 2.4	1		*qnrB16*	
	AH2014031	H	2014	–	5.7 ± 0.6	1		*qnrB76*	
	AH2014034	H	2014	±	7.0 ± 1.4	1			
	AH2014019	D	2014	–	4.1 ± 0.3	2		*qnrB92*	
	AH2014022	D	2014	^*^	4.2 ± 0.9	2			
	AH2014041	H	2014	±	6.3 ± 0.8	4		*qnrB13*	
	AH2014047	H	2014	–	3.1 ± 0.4	2		*qnrB77*	
	AH2015011	D	2015	^*^	5.5 ± 0.6	1		*qnrB77*	1
	AH2016001	D	2016	^**^	15.9 ± 0.2	2			
	AH2014030	D	2014	±	2.6 ± 1.0	1		*qnrB17*	1
	AH2014042	H	2014	±	7.2 ± 2.0	4			
	AH2016007	D	2016	–	14.3 ± 1.6	4			
	AH2014007	D	2014	^*^	14.9 ± 2.7	4		*qnrB9*	
	AH2014021	D	2014	–	3.6 ± 0.1	2		*qnrB9*	
	AH2014025	D	2014	^**^	10.3 ± 0.6	3			
	AH2014012	D	2014	–	11.1 ± 1.3	7	*bla*_CTX−M−9_		
	AH2015007	D	2015	^*^	13.4 ± 3.6	1			
	AH2014040	H	2014	±,	6.6 ± 1.2	2			
	AH2014016	D	2014	^*^	4.0 ± 0.5	1		*qnrB9*	
	AH2016013	D	2016	^*^	6.6 ± 0.9	2			
	AH2015016	D	2015	^*^	5.9 ± 1.1	2			2,3
	AH2014024	D	2014	^**^	10.4 ± 1.8	3			4
	AH2015014	D	2015	^*^	9.1 ± 0.7	1			2
	AH2014020	D	2014	^*^	4.9 ± 1.0	2			
	AH2014044	H	2014	±	7.1 ± 0.4	2			
	AH2014045	H	2014	–	5.2 ± 0.9	1			
Cluster 2	AH2015020	D	2015	–	10.5 ± 1.8	2			
	AH2015001	D	2015	^*^	6.5 ± 0.1	2			
	AH2015006	D	2015	^*^	13.4 ± 4.6	1			
	AH2015017	H	2015	^*^	22.0 ± 3.4	1			
	AH2014043	H	2014	±,	7.3 ± 0.7	3			
	AH2015005	D	2015	^***^	23.6 ± 0.7	1			
	AH2016006	D	2016	^**^	8.4 ± 2.7	1			
	AH2016004	D	2016	^**^	24.6 ± 3.0	1			
	AH2014018	D	2014	^**^	6.2 ± 1.4	3			
	AH2014046	H	2014	±	6.8 ± 2.2	3			
	AH2015008	D	2015	±	14.5 ± 5.3	1			
	AH2015012	D	2015	±	8.8 ± 1.1	1			1
	AH2015013	D	2015	±	10.5 ± 0.4	1			5
	AH2014048	H	2014	±	13.7 ± 0.3	1			
	AH2014014	D	2014	–	13.5 ± 0.4	4		*qnrS1*	
	AH2014039	H	2014	–	4.5 ± 1.4	5			
	AH2016010	D	2016	^*^	11.9 ± 1.4	2			
	AH2015009	D	2015	^*^	12.0 ± 2.9	1			1
	AH2016009	D	2016	^**^	17.8 ± 3.6	1			
	AH2015015	D	2015	^*^	8.4 ± 2.0	2			3
	AH2014015	D	2014	^**^	24.0 ± 3.1	5		*aac(6')-Ib-cr*	
Cluster 3	AH2014028	D	2014	^*^	12.8 ± 2.2	3			6
	AH2015019	H	2015	±	15.4 ± 2.2	1			
	AH2014005	D	2014	^*^	8.5 ± 0.2	3			
	AH2014008	D	2014	^*^	12.0 ± 1.7	5			
	AH2014001	D	2014	–	6.8 ± 0.6	3			
	AH2016003	D	2016	^*^	20.8 ± 0.5	1			
	AH2016005	D	2016	^**^	17.2 ± 0.2	1			
	AH2015002	D	2015	^*^	8.7 ± 2.2	4			
	AH2015004	D	2015	^**^	20.7 ± 3.0	1			
	AH2014023	D	2014	–	9.4 ± 1.0	4			7
	AH2015010	D	2015	–	4.9 ± 0.4	3			1
	AH2016015	D	2016	^**^	15.2 ± 2.2	1			
	AH2016002	D	2016	^**^	11.1 ± 1.3	2			
Cluster 4	AH2014017	D	2014	^*^	3.6 ± 0.1	3			
	AH2014027	D	2014	^**^	11.1 ± 1.5	2			5
	AH2014038	H	2014	^**^	4.8 ± 1.1	5			
	AH2016008	D	2016	^**^	16.7 ± 3.8	1			
Cluster 5	AH2014013	D	2014	^*^	13.8 ± 2.1	2			
	AH2014035	H	2014	^*^	4.9 ± 0.7	1			
	AH2014002	D	2014	–	11.0 ± 1.8	1			
	AH2014032	H	2014	^*^	3.0 ± 0.8	6	*bla*_TEM−1_	*aac(6')-Ib-cr*	
	AH2016012	D	2016	^***^	20.9 ± 2.9	1			
	AH2014003	D	2014	^*^	10.4 ± 0.8	3			
	AH2014004	D	2014	^*^	10.9 ± 0.2	3			
	AH2014036	H	2014	^**^	15.1 ± 1.1	1			
	AH2015018	H	2015	^**^	12.7 ± 2.9	1			
	AH2014006	D	2014	^**^	13.4 ± 2.5	2			
	AH2014033	H	2014	^**^	24.1 ± 3.4	1			
	AH2014029	D	2014	^*^	5.8 ± 0.2	1			8
	AH2016014	D	2016	^***^	18.0 ± 0.1	1			
	AH2014011	D	2014	^**^	12.9 ± 0.4	4			
	AH2014009	D	2014	^***^	26.6 ± 1.1	2			
	AH2014026	D	2014	^**^	27.9 ± 6.4	1			6

***, **, * *correspond to adhesion index of >50, >1 and <50 and <1 respectively. ±means ambivalent or no adhesion, –means no adhesion*.

*LDH (% ± SD): the lactate dehydrogenase released from HEp-2 cells; ST: sequence types; MDR: multidrug resistant (number of drugs resistant to); D and H: isolates from diarrheal patients and healthy individuals, respectively*.

*Other pathogens: 1, norovirus; 2, Salmonella typhimurium; 3, Aeromonashydrophila; 4, Vibrio parahaernolyticus; 5, Aeromonassobria; 6, PlesiomonasShigelloides; 7, Salmonella enteritidis; 8, Vibrio fluvialis*.

### Multi-locus sequence typing (MLST) and phylogenetic analysis

The *Citrobacter* MLST scheme (http://pubmlst.org/cfreundii/) was used. The seven housekeeping genes for MLST were *aspC, clpX, fadD, mdh, arcA, dnaG*, and *lysP*, and PCR using previously published primers and protocols (Liu et al., 2017a). The MLST primers were synthesized by Shanghai Sangon Biological Engineering Technology and Services (Shanghai, China). Sequences were analyzed using SeqMan 7.0 software.

### Antimicrobial susceptibility testing

Antimicrobial susceptibility testing was carried out using the disk diffusion method according to CLSI recommendations (Clinical and Laboratory Standards Institute, 2014). We tested the following 17 antimicrobial agents: ampicillin (AMP, 10 μg), cefotaxime (CTX, 30 μg), ceftazidime (CAZ, 30 μg), cefepime (FEP, 30 μg), cefoxitin (CFX, 30 μg), imipenem (IPM, 10 μg), aztreonam (AZM, 30 μg), cefazolin (CFZ, 30 μg), ceftriaxone (CRO, 30 μg), ciprofloxacin (CLP, 5 μg), levofloxacin (LEV, 5 μg), gentamicin (GEN, 10 μg), amikacin (AK, 30 μg), tetracycline (TET, 30 μg), chloramphenicol (CHL, 30 μg), trimethoprim/sulfamethoxazole (SXT, 25 μg) and nitrofuran (F, 300 μg) (Oxoid, Hampshire, UK). Quality control was performed using the reference *E. coli* ATCC 25922. Results were used to classify isolates as being resistant or susceptible to a particular antibiotic using standard reference values (Clinical and Laboratory Standards Institute, 2014).

### PCR amplification and sequencing

All the isolates were screened for ESBLs-encoding genes (*bla*_TEM_, *bla*_SHV_, *bla*_CTX−M−1_, *bla*_CTX−M−2_, *bla*_CTX−M−8_, *bla*_CTX−M−9_), *qnrA, qnrB, qnrS, qnrC, qnrD, aac(6')-Ib-cr*, and *qepA* by PCR using previously published primers and protocols (Liu et al., 2017a). All primers were synthesized by Shanghai Sangon Biological Engineering Technology and Services (Shanghai, China). Positive PCR products were confirmed by sequencing.

### *In vitro* adhesion and cytotoxicity assays

*In vitro* adhesion to host cells was performed using the human epidermoid carcinoma cell line HEp-2 (CCC0068; Beijing Union Medical College cell resource center), as previously described (Liu et al., 2017a). An adhesion index (<1; >1 and <50; >50) describing the mean number of bacteria per HEp-2 after examination of 10 visual fields was determined (Liu et al., 2017a). Infections were repeated three times in duplicate.

The lactate dehydrogenase (LDH) released by the HEp-2 cells was determined using the Cytotox96 kit (Promega) according to the manufacturer's instructions. The relative amount of cytotoxicity was expressed as previously described (Liu et al., 2017a). All experiments were performed three times in duplicate.

### Statistical analysis

SPSS software version 13.0 (SPSS Inc., Chicago, IL, USA) was used to conduct all statistical comparisons. A nonparametric test (Mann–Whitney U-test) was employed to compare the different groups. Two-tailed *p*-value of 0.05 or less was considered to be statistically significant.

## Results

### Multilocus sequence typing of *C. freundii* isolates

The 82 *C. freundii* isolates were divided into 76 sequence types (STs) with 65 novel STs (from ST172 to ST237), displaying high genetic diversity. No STs were predominant. Six STs each contained two isolates, of which 3 STs, ST185, ST187, and ST219, each contained two isolates with one from a diarrheal patient and one from healthy individual; two STs, ST17, and ST225 each contained two isolates from diarrheal patients; the remaining ST, ST166, had both isolates from healthy individuals.

**Figure 1:**
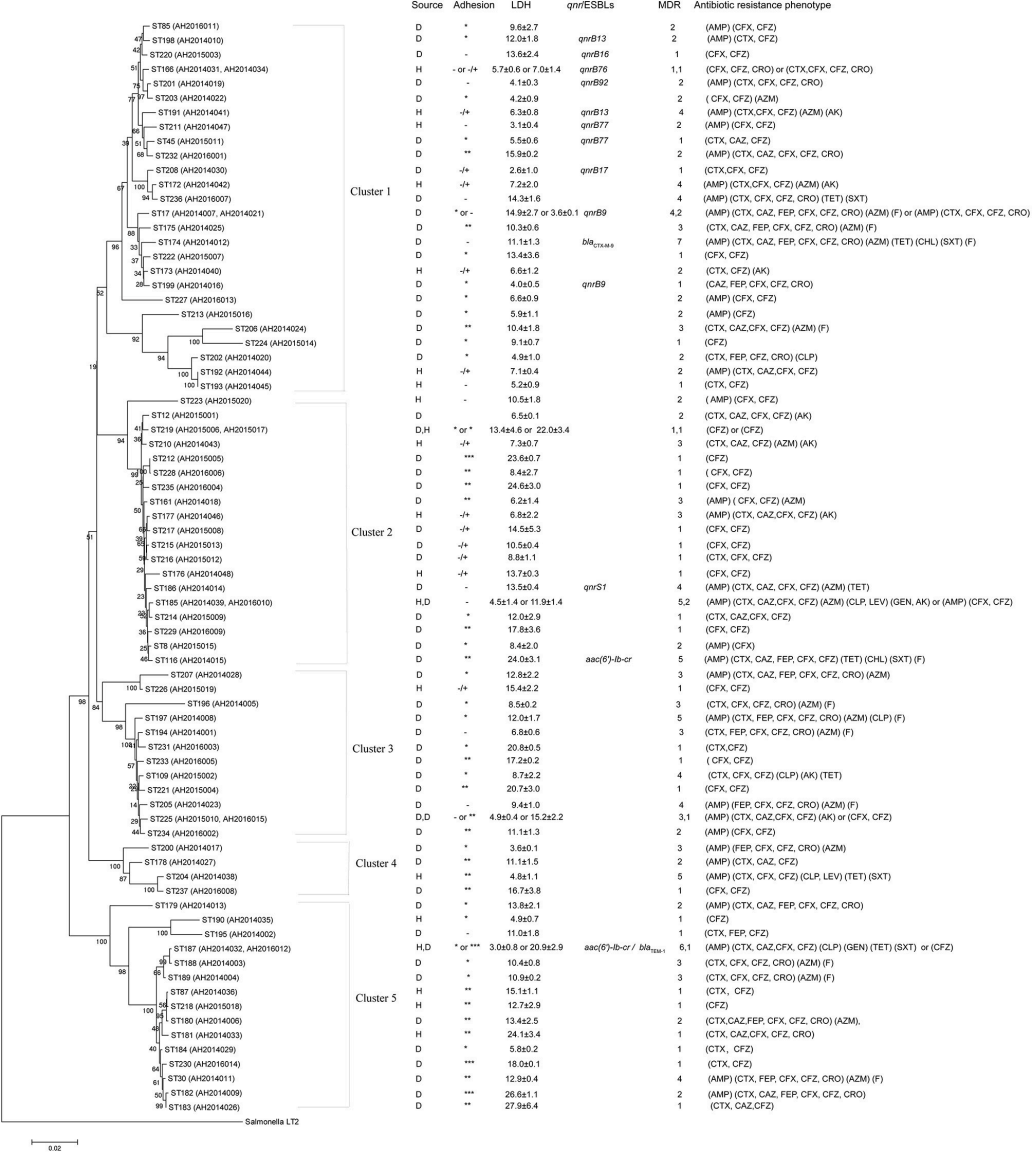
Phylogenetic relationships as determined by MLST data. The presence of ESBLs and *qnr* genes, MDR (number of drugs resistant to), adhesion, LDH and antibiotic resistance phenotype among *C. freundii* isolates were shown on the right. The tree was constructed using neighbor joining algorithm. ST, D, H, and LDH indicate sequence types, isolates from diarrheal patients and healthy individuals, and lactate dehydrogenase respectively. Cluster divisions are marked. Numbers on or near the nodes are bootstrap values from 1,000 replicates.

A phylogenetic tree for the 82 isolates was constructed using the neighbor-joining algorithm based on the concatenated sequences of the seven housekeeping genes (Figure 1). *Salmonella* LT2 was used as an outgroup. The tree could be divided into five clusters with robust bootstrap support of the major divisions. Cluster 1 was a predominant cluster containing 26 STs. *C. freundii* isolates from diarrheal patients and healthy individuals were distributed among different clusters (Figure 1).

### Prevalence of antimicrobial resistance

The 82 *C. freundii* isolates were tested for susceptibility to 17 antibiotics belonging to 10 antibiotic classes using the disk diffusion method according to CLSI recommendations (Table 2). Most of the 82 *C. freundii* isolates were resistant to β-lactams, especially to penicillins (41.5%), cephalosporins (19.5–98.8%) and monobactams (25.6%). Resistance to the two quinolones (ciprofloxacin and levofloxacin) tested was 7.3 and 2.4%, respectively; resistance to other antibiotics included aminoglycosides (2.4–11.0%), phenicols (2.4%), sulfonamides (6.1%), tetracyclines (8.5%), and nitrofuran (13.4%) (Table 2).

**Table 2:** Prevalence of resistance to different antibiotics.

**Antibiotics**	**No and % of isolates**		
	**Resistant (%)**	**Intermediary resistant (%)**	**Sensitive (%)**
**PENICLLINS**			
Ampicillin	34 (41.5)	36 (43.9)	12 (14.6)
**CEPHALOSPORINS**			
Cefotaxime	48 (58.5)	33 (40.2)	1 (1.2)
Ceftazidime	24 (29.3)	37 (45.1)	21(25.6)
Cefepime	16 (19.5)	52 (63.4)	14 (17.1)
Cefoxitin	61 (74.4)	16 (19.5)	5 (6.1)
Cefazolin	81 (98.8)	1 (1.2)	0 (0)
Ceftriaxone	23 (28.1)	33(40.2)	26 (31.7)
**MONOBACTAMS**			
Aztreonam	21 (25.6)	30 (36.6)	31 (37.8)
**CARBAPENEMS**			
Imipenem	0 (0)	7 (8.5)	75 (91.5)
**QUINOLONES**			
Ciprofloxacin	6 (7.3)	35 (42.7)	41 (50.0)
Levofloxacin	2 (2.4)	2 (2.4)	78 (95.1)
**AMINOGLYCOSIDES**			
Gentamicin	2 (2.4)	16 (19.5)	64 (78.0)
Amikacin	9 (11.0)	24 (29.3)	49 (59.8)
**TETRACYCLINES**			
Tetracycline	7 (8.5)	0(0)	75 (91.5)
**PHENICOLS**			
Chloramphenicol	2 (2.4)	5 (6.1)	75 (91.5)
**SULFONAMIDES**			
Trimethoprim/ Sulfamethoxazole	5 (6.1)	1 (1.2)	76 (92.7)
**NITROFURAN**			
Nitrofurantoin	11 (13.4)	32 (39.0)	39 (47.6)

For resistance to cephalosporins, resistance to first-generation cephalosporins, such as cefazolin and second-generation cephalosporins, such as cefoxitin, were 98.8 and 74.4%, respectively; less common was resistance to ceftriaxone, ceftazidime, and cefepime with prevalence of 28.0, 29.3, and 19.5%, respectively (Table 2).

Twenty-six isolates were multidrug resistant (MDR), with resistance to at least one antibiotic of three or more distinct classes (MDR ≥ 3). Of the 26 MDR isolates, 23 were isolated from 2014, two were from 2015 and one were from 2016 (Table 1).

The 26 MDR isolates were distributed in 5 clusters, and mainly in cluster 1 and 3 which included 7 MDR isolates, respectively (Figure 1 and Table 1).

The genes *bla*_CTX−M−9_*, bla*_TEM−1_*, aac(6')-Ib-cr, qnrS1, qnrB9, qnrB13, qnrB16, qnrB17, qnrB76, qnrB77*, and *qnrB92* were detected in 15 of the 82 *C. freundii* isolates. Five MDR isolates, all of which were isolated from 2014, harbored the genes *bla*_TEM−1_, *bla*_CTX−M−9_, *aac(6')-Ib-cr, qnrS1, qnrB9, and qnrB13*, respectively (Figure 1 and Table 1).

Two ESBLs isolates (AH2014012 and AH2014032) and five of six fluoroquinolones resistant isolates (Table 3) were MDR.

**Table 3:** Fluoroquinolone and extended-spectrum-L-lactams (ESBLs) resistant strains.

**Isolates**	**Year**	**STs**	**MDR**	**Antibiotic resistance phenotype**	**ESBLs**	***Qnr***
AH2014008	2014	197	5	(AMP) (CTX, FEP, CFX, CFZ, CRO) (AZM) (CLP) (F)		
AH2014020	2014	202	2	(CTX, FEP, CFZ, CRO) (CLP)		
AH2014032	2014	187	6	(AMP) (CTX, CAZ, CFX, CFZ) (CLP) (GEN) (TET) (SXT)	*bla*_TEM−1_	*aac(6')-Ib-cr*
AH2014038	2014	204	5	(AMP) (CTX, CFX, CFZ) (CLP, LEV) (TET) (SXT)		
AH2014039	2014	185	5	(AMP) (CTX, CAZ, CFX, CFZ) (AZM) (CLP, LEV) (GEN, AK)		
AH2015002	2015	109	4	(CTX, CFX, CFZ) (CLP) (AK) (TET)		
AH2014012	2014	174	7	(AMP) (CTX, CAZ, FEP, CFX, CFZ, CRO) (AZM) (TET) (CHL) (SXT) (F)	*bla*_CTX−M−9_	

Mutations of quinolone resistance-determining regions (QRDRs) of *gyrA* and *parC* genes were screened by PCR sequencing in ciprofloxacin resistant strains. Four (AH2015002, AH2014032, AH2014038, and AH2014039) of the six ciprofloxacin resistant strains (AH2014008, AH2014020, AH2015002, AH2014032, AH2014038, and AH2014039) showed mutations in codons 59, 111, and/or 134 in the QRDR region of the *gyrA* gene: Thr59Ile in AH2015002, AH2014032, and AH2014039, and Thr59Ile, Gln111Arg, and Ile134Val in AH2014038. None of the six ciprofloxacin resistant strains had any mutations in the QRDR region of the *parC* gene.

By phylogenetic clusters (Figure 1 and Table 1), cluster 1, cluster 2, and cluster 5 contained *C. freundii* isolates carrying ESBLs, *qnr* or *aac(6')-Ib-cr* genes, and cluster 1 contained all *qnrB*- carrying isolates.

### Prevalence of *qnrB* genes

Eleven *C. freundii* isolates were found to harbor *qnrB* genes. Sequence analysis revealed that three isolates (AH2014007, AH2014016, and AH2014021) harbored an identical *qnrB* sequence (*qnrB9)*, AH2014010 and AH2014041 harbored *qnrB13*, AH2014047 and AH2015011 harbored *qnrB77*, moreover, AH2015003, AH2014030, and AH2014031 harbored *qnrB16, qnrB17*, and *qnrB76*, respectively.

One isolate (AH2014019) was found to harbor a new *qnrB* gene. Sequence analysis revealed that it differed from the *qnrB76* gene by two nucleotide changes (GenBank accession no.KM985469.1). One, a T → G change at nt469 resulted in Ser → Ala, while the other, a C → T change at nt391 was synonymous. Hence this new *qnrB* allele is designated as *qnrB92* (GenBank accession no. MG744557), a new variant of the *qnrB* gene, in accordance with the *qnr* nomenclature rules of Jacoby et al. (2008).

Two of the 11 *qnrB* isolates were MDR ≥ 3. All 11 *qnrB* isolates were susceptible to ciprofloxacin and levofloxacin.

### Adherence of *C. freundii* isolates

We tested the 82 isolates for adhesion to HEp-2 cells and categorized the extent of adhesion using the adhesive index (Table 1; Mange et al., 2006). Four *C. freundii* isolates showed the strongest adhesion, with an adhesion index greater than 50. Twenty-one isolates showed intermediate adhesion, with an adhesion index between 1 and 50. Twenty-nine isolates showed little adhesion, with an adhesion index of less than one. The remaining isolates showed ambivalent adhesion or no adhesion.

By phylogenetic clusters (Figure 1 and Table 1), the majority of the isolates (9/16) in cluster 5 showed intermediate or strong adhesion, while cluster 1 was the opposite with the majority of the isolates (25/28) showing little or no adhesion. All strongest adhesive isolates belonged to cluster 2 or 5.

### Cytotoxicity of *C. freundii* isolates

**Figure 2:**
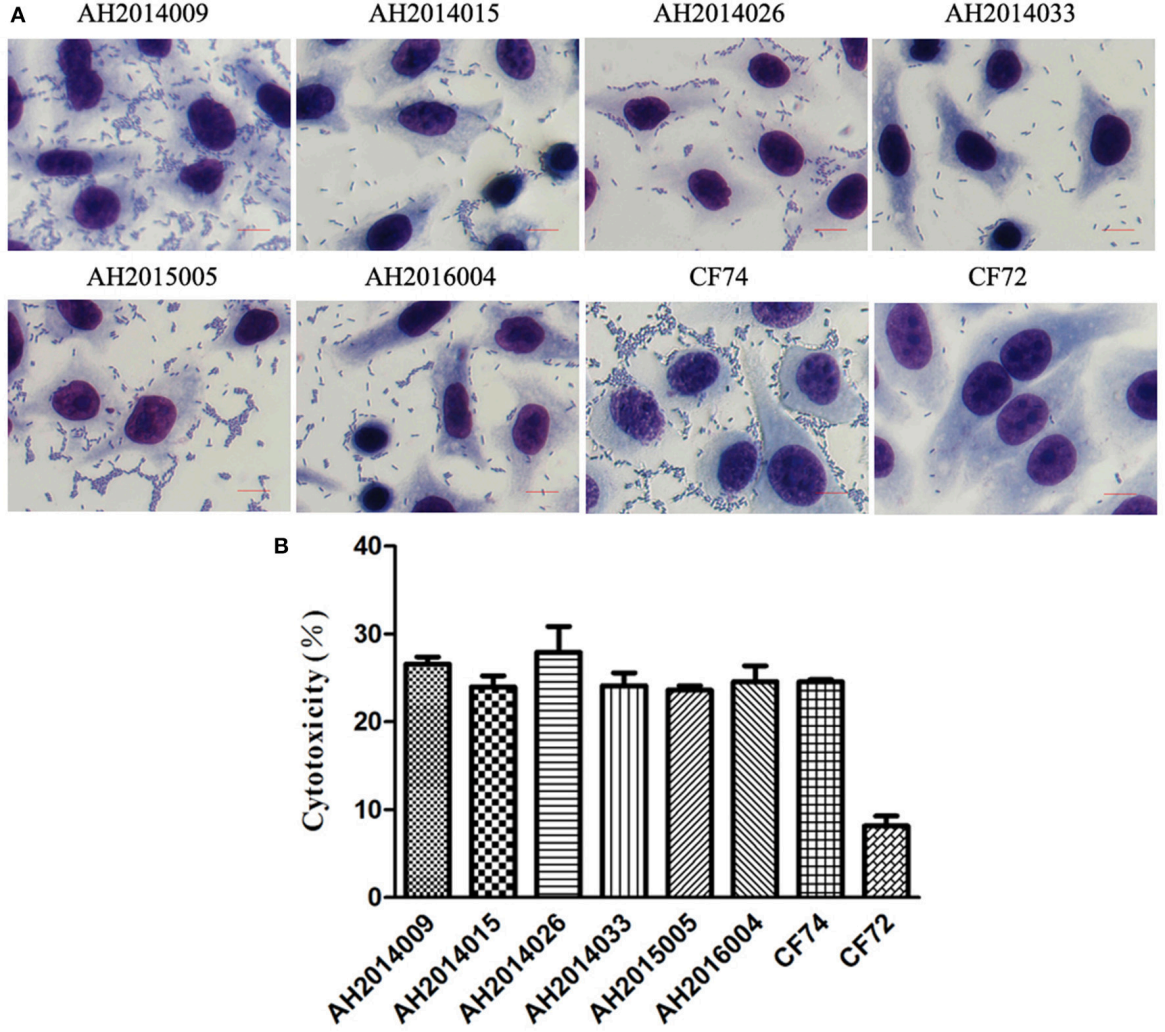
HEp-2 cell adhesion and cytotoxicity of *C. freundii* isolates. **(A)** Light micrographs show the adherence patterns displayed by six strongly cytotoxic *C. freundii* isolates, CF74 and CF72. Bar: 10 μm. **(B)** Cytotoxicity was based the LDH released from HEp-2 cells after exposure to six strongly cytotoxic *C. freundii* isolates at 8 h. CF72 and CF74 were control strains.

The 82 *C. freundii* isolates were tested for cytotoxicity to cultured HEp-2 cells by measuring the amount of lactate dehydrogenase (LDH) released by HEp-2 cells. The released LDH levels ranged from 3.0 to 27.9% (Table 1). *C. freundii* strains CF74 and CF72 were used as positive and negative controls of cytotoxicity respectively (Liu et al., 2017a). The levels of LDH released by CF74 and CF72 were 24.5 and 8.1%, respectively. Six isolates released LDH more than 24%, showing high cytotoxicity (Table 1). Among these six isolates, two isolates showed strongest adherence while other four isolates showed intermediate adhesion (Table 1 and Figure 2). Another five isolates released LDH from 18.0 to 22.0% and are considered intermediate cytotoxic. The remaining 71 isolates showed LDH release less than 17.8% and are likely to be non-cytotoxic (Table 1).

One highly cytotoxic isolate was MDR with resistance to five antibiotics (penicillins, cephalosporins, tetracyclines, phenicols, and sulfonamides) and harbored an *aac(6')-Ib-cr* gene. The other five highly cytotoxic isolates were resistant to fewer than 3 antibiotic classes.

By phylogenetic clusters (Figure 1 and Table 1), cluster 1 was least cytotoxic on average (8.0 ± 3.9) while cluster 5 was the most cytotoxic (14.5 ± 7.4). The difference between cluster 1 and cluster 5 is statistically significant (*p* < 0.01). Clusters 2 and 3 were similarly cytotoxic (12.3 ± 2.0 and 12.6 ± 1.4) and were also significantly higher than cluster 1 isolates (*p* < 0.05). Two and three high cytotoxic *C. freundii* isolates belonged to cluster 2 and cluster 5, respectively.

## Discussion

*C. freundii* is a recognized emerging opportunistic pathogen and has been implicated in gastroenteritis and foodborne outbreaks (Ifeadike et al., 2012; Settanni et al., 2013). A most recent foodborne outbreak reported in Germany was caused by a novel ST (Pletz et al., 2018). In this study, we examined 82 isolates of *C. freundii* obtained from 62 diarrheal patients from the majority of whom no other pathogens were isolated and also from 20 healthy individuals to assess the genetic diversity and antimicrobial resistance and *in vitro* virulence phenotypes.

The 82 isolates were separated into 76 STs and 5 phylogenetic clusters. The 76 STs from this study were compared with 171 STs from the *Citrobacer* MLST database, We found that 11 STs in this study shared the same sequence types with isolates from the database from other countries or regions or from different sources. Among these 11 STs, ST8 contained isolates from the urine of an acute myeloid leukemia patient from Poland (Majewski et al., 2017); ST12 contained isolates from a rectal swab; ST17 contained isolates from skin necrosis, urine and rectal swabs; ST30 contained isolates from fecal samples of diarrheal patients in our previous study (Liu et al., 2017a); ST87, ST85, and ST45 each contained isolates from food in our previous study (Liu et al., 2017a; ST116 contained isolates from blood; ST161 and ST166 contained isolates from water. Therefore strains of the same STs of *C. freundii* may be widely present in fecal, food, and other reservoirs.

In this study, we analyzed isolates from both diarrheal patients and healthy individuals in an attempt to further understand the genetic diversity of potential diarrheagenic *C. fruendii*. Majority of the diarrheal cases had no other pathogens isolated with *C. freundii* being possibly the causal organism. However, there is no separation of isolates from clinical cases and healthy individuals. Some of the diarrheal patients also had other pathogens isolated. There was no distinction of the isolates from these different sources by STs, phylogenetic clusters or adhesion/cytotoxicity phenotypes. Among the 82 *C. freundii* isolates we obtained from diarrheal outpatients and healthy individuals, 25 (30.5%) of the isolates showed moderate to strong adhesion. Among these 25 adhesive isolates, 9 isolates showed moderate to strong cytotoxicity, indicating their pathogenic potential. Interestingly, adhesiveness and cytotoxicity were clustered by phylogenetic clusters. Nine (56%) and five (31%) of the 16 isolates in cluster 5 showed intermediate/strong adhesion and intermediate/high cytotoxicity, respectively. In comparison, only three (11%) and none (0%) of the 28 cluster 1 isolates were adhesive or cytotoxic respectively. All strongest adhesive and highly cytotoxic isolates belonged to cluster 2 or 5. Clearly there is a difference in virulence between clusters by the measures of *in vitro* virulence properties. However, both cluster 1 and cluster 5 are similarly likely to be isolated from healthy individuals with 8 of the 28 and 5 of the 16 isolates respectively. Further work is required to determine their difference in pathogenicity and disease.

*C. freundii* has become increasingly resistant to a range of antibiotics (Liu et al., 2017b). Liu et al. (2017b) reported that blood isolates of *C. freundii* from hospital in Taiwan showed a high rate of resistance (66.7–97.2%) to second-generation cephalosporin and cephamycin. Mohanty et al. (2007) reported that isolates of *C. freundii* isolates from patients in a tertiary care hospital of India had high degrees of resistance to ceftazidime (85%), cefotaxime (85%), piperacillin (65%), and ciprofloxacin (60%). In our study, 98.8% of the isolates were resistant to first-generation cephalosporins, such as cefazolin, 74.4% resistant to second-generation cephalosporins, such as cefoxitin, 28–29.3% resistant to third-generation cephalosporins and 19.5% resistant to fourth-generation cephalosporins.

*C. freundii* is often resistant to multiple classes of antibiotics, suggesting that both clinical and environmental strains may be a reservoir of antimicrobial resistance determinants (Pepperell et al., 2002; Gupta et al., 2003; Nada et al., 2004; Yim et al., 2013; Feng et al., 2015; Leski et al., 2016a; Sheppard et al., 2016). MDR *C. freundii* strains have been associated with a higher rate of in-hospital mortality compared to susceptible strains (Leski et al., 2016b). A survey of outpatients in Sierra Leone revealed that *C. freundii* isolates from UTIs were highly MDR with 22 isolates resistant to >7 antibiotics out of the 11 tested, and 81.8% of the isolates produced ESBLs (Leski et al., 2016b). In this study, we surveyed *C. freundii* from diarrheal outpatients, 30.6% isolates were resistant to ≥3 antibiotic classes out of the 10 distinct antibiotic classes tested. One MDR isolate was strongly cytotoxic. Such highly cytotoxic MDR strains may cause more severe disease and their MDR properties may limit clinical therapeutic options when they cause disease.

ESBLs in *C. freundii* have been widely reported (Fernandes et al., 2014; Liu et al., 2017b). In 36 blood *C. freundii* isolates from a Taiwanese hospital, 16.7% of the isolates carried the *bla*_TEM−1_ gene and 5.6% carried *bla*_SHV−12_ or *bla*_CTX−M−15_ (Liu et al., 2017b). In this study, we did not test for ESBL phenotype but screened by PCR for *bla*_CTX−M_, *bla*_TEM_ and *bla*_SHV_ genes. We found one isolate each *bla*_CTX−M−9_ and *bla*_TEM_ positive, but none *bla*_SHV_ positive (Table 3).

The prevalence of *qnr* and *aac(6')-Ib-cr* genes varied (Liu et al., 2017a). In China, Yang et al. (2008) had repored that the prevalence of *qnr* and *aac(6')-Ib-cr* genes in *C. freundii* isolates from southern China (including Shanghai, Wuhan, Nanjing, Guangzhou, and Fuzhou) and northern China (including Beijing, Tianjin, Shenyang, and Jinan) was at 63.3 and 26.7% respectively; while Zhang et al. (2012) showed the prevalence of *qnr* and *aac(6')-Ib-cr* in *C. freundii* from southern China (Hangzhou) was at 72.8 and 68.9% respectively. In Korea, Park et al. (Park et al., 2007) showed that 38.4% of *C. freundii* isolates harbored *qnr* determinants. In our previous study (Liu et al., 2017a), we found much lower prevalence of *qnr* and *aac(6')-Ib-cr* genes in *C. freundii* isolates at 23.1 and 15.4% respectively. In the present study, we also found low prevalence of *qnr* and *aac(6')-Ib-cr* genes in *C. freundii* isolates at 14.6 and 2.4% respectively. Our isolates for both studies were from south central region of China (Maanshan city, Anhui Province). These findings suggest regional difference in the prevalence of resistance within a country as well as between countries.

The *qnrB* genes constitute the most prevalent and diverse group within the *qnr* family, encoding proteins responsible for decreased susceptibility to fluoroquinolones (Jacoby et al., 2011; Ribeiro et al., 2015). We found a new *qnrB* gene, designated as *qnrB92* (GenBank accession no. MG744557) in one *C. freundii* isolate. However, *qnrB*-carrying *C. freundii* isolates do not always show high level of quinolone resistance (Zhang et al., 2012; Liu et al., 2017a). Our results were consistent with this observation. The new *qnrB92*-carrying *C. freundii* isolate was susceptible to ciprofloxacin and levofloxacin and indeed none of the 11 *qnrB* isolates was resistant to ciprofloxacin and levofloxacin. However, two *qnrB*-carrying *C. freundii* isolates we previously reported had a high MIC for NAL (>128 μg/mL) (Liu et al., 2017a). *C. freundii* isolates carrying *qnrS* and *aac(6')-Ib-cr* have been shown to have a higher MIC for quinolones (Zhang et al., 2012). In our previous study, we found that one *aac(6')-Ib-cr*-carrying *C. freundii* and one *qnrS1*-carrying *C. freundii* isolates had high MIC of three quinolones (NAL, >128 μg/mL; CLP, >32 μg/mL; LEV, >16 μg/mL) (Liu et al., 2017a). However, in this study, two *aac(6')-Ib-cr*-carrying *C. freundii* (AH2014032 and AH2014015) and one *qnrS1*-carrying *C. freundii* (AH2014014) isolates were resistant or intermediary resistant to ciprofloxacin, but susceptible to levofloxacin. Isolate AH2014032 showed a QRDR region with the mutation of Thr59Ile of the *gyrA* gene and no mutation in the *parC* gene, while AH2014015 and AH2014014 did not carry any mutations in *gyrA* and *parC* genes.

Resistance to ciprofloxacin was lower in both our previous study and this study at 13.0% (8/62) and 7.3% (6/82) respectively. This difference may be regional. There seems to be regional difference in the prevalence of *qnr* and *aac(6')-Ib-cr* genes as discussed above. Our studies were conducted in one region of China which may differ in the prevalence of ciprofloxacin resistance from other regions in China and from other countries (Park et al., 2007; Yang et al., 2008; Zhang et al., 2012). It is also possible that the low prevalence was due to the source of the isolates. Isolates for our previous study and for this study were from food and from outpatients respectively, while the high rate reported in India was from isolates from hospitalized patients (Mohanty et al., 2007).

## Conclusion

We isolated 82 *C. fruendii* isolates from human diarrheal outpatients and healthy individuals in Maanshan, Anhui Province, China and found a high diversity of the isolates by sequence types with 65 STs being novel. Eleven STs were found in the MLST database which contained isolates from different sources and/or geographic regions. The isolates varied in *in vitro* virulence phenotypes (adhesion and cytotoxicity) with most isolates in phylogenetic cluster 5 being adhesive and cytotoxic. Prevalence of MDR of three or more antibiotic classes out of the 10 distinct antibiotic classes tested was at 31.7%. Each of the *bla*_CTX−M−9_, *bla*_TEM−1_, *qnrS1* and *aac(6')-Ib-cr* genes was detected in one *C. freundii* isolate*. Six* isolates that showed strong cytotoxicity to HEp-2 cells, one of which was multidrug resistant. We also found a new *qnrB* gene (*qnrB92*) in one *C. freundii* isolate. This study has shed more light on the genetic diversity, pathogenicity and antibiotic resistance of *C. fruendii*.

## Author contributions

LiyL and JX: designed the project; DC: carried out the sampling work; WJ, HS, SH, and YL: carried out the experiments; LiyL, YW, and LiqL: analyzed data; LiyL and RL: drafted the manuscript. All authors have read and approved the final version of the manuscript.

### Conflict of interest statement

The authors declare that the research was conducted in the absence of any commercial or financial relationships that could be construed as a potential conflict of interest.
